# Dosimetric accuracy of tomotherapy dose calculation in thorax lesions

**DOI:** 10.1186/1748-717X-6-14

**Published:** 2011-02-09

**Authors:** Veronica Ardu, Sara Broggi, Giovanni Mauro Cattaneo, Paola Mangili, Riccardo Calandrino

**Affiliations:** 1 Medical Physics Department, IRCCS San Raffaele, Milano, Italy

## Abstract

**Background:**

To analyse limits and capabilities in dose calculation of collapsed-cone-convolution (CCC) algorithm implemented in helical tomotherapy (HT) treatment planning system for thorax lesions.

**Methods:**

The agreement between measured and calculated dose was verified both in homogeneous (Cheese Phantom) and in a custom-made inhomogeneous phantom. The inhomogeneous phantom was employed to mimic a patient's thorax region with lung density encountered in extreme cases and acrylic inserts of various dimensions and positions inside the lung cavity. For both phantoms, different lung treatment plans (single or multiple metastases and targets in the mediastinum) using HT technique were simulated and verified. Point and planar dose measurements, both with radiographic extended-dose-range (EDR2) and radiochromic external-beam-therapy (EBT2) films, were performed. Absolute point dose measurements, dose profile comparisons and quantitative analysis of gamma function distributions were analyzed.

**Results:**

An excellent agreement between measured and calculated dose distributions was found in homogeneous media, both for point and planar dose measurements. Absolute dose deviations <3% were found for all considered measurement points, both inside the PTV and in critical structures. Very good results were also found for planar dose distribution comparisons, where at least 96% of all points satisfied the gamma acceptance criteria (3%-3 mm), both for EDR2 and for EBT2 films. Acceptable results were also reported for the inhomogeneous phantom. Similar point dose deviations were found with slightly worse agreement for the planar dose distribution comparison: 96% of all points passed the gamma analysis test with acceptable levels of 4%-4 mm and 5%-4 mm, for EDR2 and EBT2 films respectively. Lower accuracy was observed in high dose/low density regions, where CCC seems to overestimate the measured dose around 4-5%.

**Conclusions:**

Very acceptable accuracy was found for complex lung treatment plans calculated with CCC algorithm implemented in the tomotherapy TPS even in the heterogeneous phantom with very low lung-density.

## 1. Introduction

Image-guided intensity modulated radiation therapy (IG-IMRT) techniques are becoming more popular due to the possibility to create and monitor escalated dose distributions highly conformed to irregular-shaped targets. The implementation of such new technology requires a precise and accurate dose calculation algorithm which can generate reliable dose distributions and dose-volume information for treatment planning calculation and evaluation.

An ideal dose calculation algorithm should take into account relative electron density and dimensions of inhomogeneous media, electronic disequilibrium for high energy photon beams and electron transport at interfaces between media of different densities [1]. Monte Carlo (MC) simulation is well known as the most accurate algorithm for dose calculation in the presence of inhomogeneous media [2-4]. However, other semi-empirical dose calculation algorithms are generally clinically implemented and used in the treatment planning systems [5,6]. Convolution/superposition models are now commonly used in treatment planning systems [7-9]. Although they present major improvements compared to older pencil beam algorithms [10] due to empirical approximations, they may introduce appreciable inaccuracies in the dose distributions, especially in case of small or superimposed small fields (typically found in IMRT treatments) irradiating low density media; comparing the collapsed cone convolution approach to MC, Chow et al [11] reported significant dose deviation with 6 MV photon beam when the electron density is less than 0.3 and small field sizes are used. Fogliata et al [12] investigated the influence of different air filling in lungs on the calculation accuracy of photon dose algorithms compared with MC: with a 6 MV photon beam, all the investigated algorithms had a peak of failures for densities of the order of 0.05 g/cm^3^.

Due to the rapid evolution of the available treatment techniques, irregular fields and steep dose gradients are applied in order to achieve highly conformal dose distributions; under these conditions high dosimetric accuracy of any IMRT treatment planning system is of crucial importance for the effectiveness and success of the treatment prescribed [13].

The aim of this paper was to investigate the dose calculation accuracy in (very) low-density lung media for treatments delivered by a Helical Tomotherapy unit (HT), where the calculation dose is performed using a convolution-superposition algorithm (C/S) based on a collapsed cone (CCC) approach [14-16]. The CCC superposition (CCC/S) dose algorithm has been shown to accurately predict dose distributions for IMRT techniques, including helical tomotherapy, although most published results refer to water equivalent phantom with simple geometries.

Several papers [17-20] have investigated the accuracy of the CCC/S dose algorithm implemented in HT treatment planning in case of inhomogeneous tissues for some limited cases. Chaudhari et al [17] analyzed only two clinical esophageal cancers simulated in a custom-designed heterogeneous phantom mimicking the mediastinum geometry by considering two different lung-equivalent materials with density equal to 0.28 g/cm^3 ^and 0.16 g/cm^3^, respectively.

Zhao et al [18] investigated the accuracy of the algorithm by considering only one clinical lung treatment delivered on a CIRS (Computerized Imaging Reference Systems, Inc) anthropomorphic heterogeneous phantom, where dose distributions calculated from HT treatment planning were compared both with measurements and with MC calculations. Also in the Sterpin et al paper [20] the CCC/S algorithm implemented in the HT unit was compared with MC simulations only for small lung tumors with diameter <3 cm.

In this work we focused our analysis by simulating some thorax treatments (mediastinal lesions, single or multiple metastasis) of different geometries. For the considered cases, the dose calculation algorithm accuracy was investigated in both a homogenous (15 plans) and inhomogeneous (4 plans) phantom (where the lungs consisted of material with a density equal to 0.04 g/cm^3^) by absolute ionization dose measurements, dose profile comparisons and quantitative analysis of dose distributions. A comparison between dose distributions measured on EDR2 and EBT2 films was also reported.

## 2. Materials and methods

### 2.1 Phantoms design

Measurements were performed in both a homogeneous and a custom-made heterogeneous phantom mimicking a patient's thorax region.

As homogeneous phantom we used the Cheese Phantom, typically employed in our clinic for routine patient QA (DQA) measurements. It is a solid water cylindrical phantom of 15 cm radius and 18 cm length cut into two semi-cylindrical halves to allow the insertion of a film along the central plane. Along the other direction a series of holes, interspaced by 1 cm (one hole is set 0.5 cm from the central plane of the film), allows the insertion of ionization chambers for point measurements. Film and chamber measurements can be performed at the same time by considering both the sagittal and coronal plane. In this paper for all simulated plans the film was set along the coronal plane and the absolute ionization measurements were performed in points along the sagittal direction.

**Figure 1:**
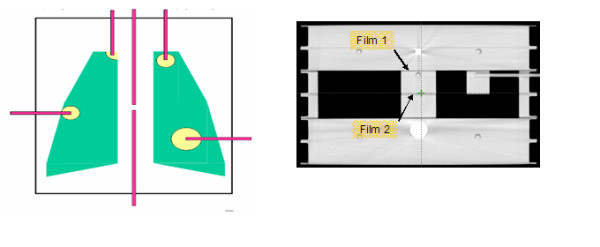
**Heterogeneous thorax phantom**. **1a)** Six slabs of acrylic (density 1.16 g/cm^3^) simulating the homogeneous media, with an aluminum cylindrical insert (2.7 g/cm^3^) simulating bone equivalent material and two low density (0.04 g/cm^3^) inserts symmetrically positioned and separated by an acrylic area (mediastinum), simulating lung region. **1b)** For film measurements film 1 is positioned between the second (homogeneous slab) and the third slab (inhomogeneous slab); film 2 is positioned between the two inhomogeneous slabs with lung media.

A custom designed phantom mimicking the patient's thorax region was defined (Figure 1a). It is composed of six slabs of 30 cm × 40 cm × 3 cm of acrylic (density 1.16 g/cm^3^) simulating the homogeneous media. Three slabs, two positioned on the top and one on the bottom of the phantom were completely homogeneous; inside one homogeneous slab an aluminum cylindrical insert (2.7 g/cm^3^) was considered. The other two slabs simulate the lung region using Styrofoam: two low density (0.04 g/cm^3^) inserts were symmetrically positioned and separated by an acrylic area (mediastinum). Fogliata et al [12], showed that the lung mass density varies during respiratory phases; in free breathing and in deep inspiration breath hold the mean densities are 0.27 and 0.16 g/cm^3 ^respectively with peak densities of 0.17 and 0.09 g/cm^3 ^.

Inside lung volumes, acrylic inserts of various dimensions and positions, simulating the tumor lesions (metastasis), were positioned. They are cylindrical with a radius of 1, 2 or 3 cm, positioned completely inside or in the boundary of the lung; these different geometries are useful to simulate several clinical situations. The phantom was designed in order to allow both planar and point dose measurements. Films can be placed along horizontal planes between the different slabs; absolute point dose measurements can be performed both in all tumor inserts and in the homogeneous mediastinum region, thanks to several inserts created inside the phantom.

### 2.2 Treatment planning

For homogeneous phantom measurements, specific DQA plans of fifteen patients (pts) previously treated for lung tumour using the Helical Tomotherapy technique were created. The treatment volumes considered can be divided into three groups: mediastinal lesions (9 pts), single lung metastasis (2 pts), multiple lung metastases (4 pts). Single and multiple metastases were treated based on a hypofractionated approach with 9 Gy of daily dose; different fractionated regimes (2 Gy/day; 2.5 Gy/day, 4 Gy/day) were applied for mediastinal tumours.

All plans were generated using a 25 mm field width, a pitch equal to 0.287 for conventional fractionation or in the range of 0.2-0.3 for hypofractionated regimes and a modulation factor of approximately 2.5 -3.

**Figure 2:**
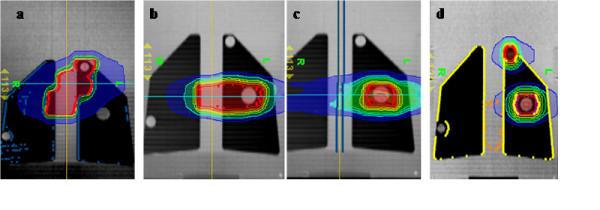
**Coronal dose distributions of the four treatment plans generated on the thorax heterogeneous phantom: mediastinic lesions, Med1(2a) and Med2 (2b), single metastasis (2c) and multiple metastasis plan (2d)**.

In all patient treatment plans considered, the aim of the optimisation process was the homogeneous coverage of the PTV, concomitant with organ at risks (spinal cord, heart, lung, oesophagus) sparing. For the heterogeneous phantom, four treatment plans were generated simulating four different clinical volumes: a single lung metastasis, multiple lung lesions and two different mediastinic target volumes; two different mediastinic targets (Med1 and Med2) were considered with two different volumes and with a different target portion in the lung region. Doses and planning parameters used in our clinical practice were adopted for these treatment planning simulations. Coronal dose distributions for each hetereogeneous plan are shown in Figure 2.

### 2.3 Film and ionization chamber dosimetry

Radiographic (Kodak EDR2) and radiochromic (Gafchromic EBT2) films were used for planar measurements. In both cases a calibration curve was created to correlate the measured film's optical density with the delivered dose, irradiating the film with a static uniform field at 5 cm depth; two sensitometric curves in the dose range from 0.12 Gy to 6.88 Gy for EDR2 films and between 0.12 Gy and 8 Gy for EBT2 respectively were created. Different calibration curves were created for each films batch used.

A commercial Vidar film digitizer (DosimetryPro Advantage, Vidar Systems Corp., Herndon, VA) was used to scan EDR2 films.

Gafchromic EBT2 films were scanned with EPSON Pro V750 Expression scanner A4 size at least 10 h after irradiation [21]. The software package "EPSON scan" (professional mode with all image adjustments and colour corrections turned off) was used to scan and acquire images. Films were scanned in the 48 bit red-green-blue (RGB) mode with a resolution of 72 dpi. A median filter (3 × 3) was applied to reduce noise. Data were saved in a tagged image file (TIFF). Film sheet orientation was maintained in the centre of the scan to guarantee better response stability. A correction matrix dependent on the pixel position and the different dose levels was applied in order to manage the light scattering of the scanner lamp and its non- uniform response [22].

For absolute point dose measurements, an Exradin A1SL ion chamber (Standard Imaging, Middleton, WI) was used. The A1SL has a small volume of 0.056 cm^3^, which makes it a good candidate for point dose measurements. The absolute dose was defined according to the International Atomic Energy Agency's (IAEA) recommended absolute dosimetry protocol (TSR 398) applying appropriate correction factors for beam quality and environmental conditions [23].

### 2.4 DQA procedure

A patient specific DQA plan was generated for each treatment plan by considering the export of the treatment's fluence and the dose distribution recalculation, both on the homogeneous phantom (Cheese Phantom) and on the heterogeneous thorax phantom.

For each DQA plan, film and ion chamber measurements were taken in order to verify the agreement between measured and calculated dose distributions, both for absolute dose points and for planar dose distribution.

For homogeneous DQA plans, films (EDR2 and EBT2) were set in the coronal plane with concomitant point dose measurements in the sagittal direction.

To minimize chamber position uncertainty, dose measurements points were selected in the high dose/low gradient or low dose/low gradient regions; absolute dose measurements were performed in 22 points inside the high dose/low gradient PTV region (15 points for mediastinic lesion, 4 and 3 points for single and multiple metastasis, respectively) and in 27 points (15 for mediastinic lesions, 7 for single and 5 for multiple metastasis) inside the low dose/low gradient OAR structures.

Relative EDR2 film dose distributions were normalised to the absolute dose measured with ionization chamber in the PTV points, proximal to the film's coronal plane; EBT2 absolute dose distributions were considered.

Similar procedures were performed for the DQA plans in the heterogeneous thorax phantom. Obviously in this case, treatment plan and relative DQA plan haven't any differences, due the same thorax inhomogeneous phantom was used to simulate inhomogeneous treatment plans and for DQA measurements. For each DQA plan two to four absolute dose points were acquired, both in the high dose/low gradient PTV region and in the low dose region corresponding to critical structures or healthy tissue. Two films were used for each DQA plan: the first (reported in the text as Film1) was placed in the interface region between a homogeneous slab and the low density lung slab, the second (Film 2) between the two slabs with the low density lung inserts (Figure 1b). Similarly to homogeneous measurements, relative EDR2 film dose distributions were normalised to the absolute dose measured with the ionization chamber; EBT2 films were used in a relative way by normalising both measured and calculated dose distributions in a point inside the PTV region.

### 2.5 Data analysis

The agreement among measured and calculated dose distributions was evaluated in terms of percentage difference between absolute point dose measurements, qualitative dose profile comparisons and a quantitative analysis of dose distribution through gamma function analysis [24]. For the point dose measurement the percent discrepancy was calculated according to: %Δ = 100*(Dm-Dc)/Dc, where Dm is the measured point dose and Dc is the calculated dose at the same position.

The γ - map analysis is a method that conjugates both the dose difference (ΔDD) and the distance to agreement (ΔDTA) pass/fail criteria. The planar map of γ values gives a qualitative representation of the agreement of two distributions; a quantitative evaluation could be defined based on the analysis of γ -area histograms, defining the percentage of γ -values below a certain threshold.

Profiles and dose map comparisons [13] were performed using TomoTherapy Inc. software. We quantitatively analysed the gamma function by considering the γ-area histograms and distribution using the Tomotherapy Inc software (Research station), by considering all the points of the film that are included in the homogeneous/inhomogeneous phantoms. In our analysis the dose difference criteria is defined respect to the prescribed dose calculated in the DQA dose distribution (the calculated dose distribution exported on the phantom).

Different acceptance criteria were used for γ analysis: 3%-3 mm and 4%-3 mm for the homogeneous phantom; 3%-3 mm, 4%-3 mm, 4%-4 mm and 5% -4 mm for the heterogeneous thorax phantom.

In the clinical practice we consider as acceptance criteria ΔDD = 3% and ΔDTA = 3 mm in case of simple case as spherical lesions and without stressing modulated dose distributions; ΔDD = 4% and ΔDTA = 3 mm in case of more complex geometries including irregular-shaped targets, proximity of critical OARs to spare and then dose distributions with very high and deep dose gradients. We were confident that these criteria agree with those suggested in the ESTRO Booklet n°7 [25]

## 3. Results

### 3.1 Homogeneous phantom

Absolute point measurements are shown in Table 1, where the average percent discrepancy between measured and calculated dose is reported, respectively for PTV points (22 points) (high dose/low gradient dose points) and for critical structure regions (27 points) (high dose/high gradient, low dose/low gradient dose points), by considering, separately, the three anatomical districts. Excellent agreement (< 2%) between measured and calculated dose was found: an overall average discrepancy equal to 0.7% (1SD = 1.2%) and to 1% (1SD = 0.4%) was found for PTV and for OARs respectively. The largest average difference (1.9%) was found for single metastasis treatment plans, possible due to the more critical positioning in small target volumes.

**Table 1:** Ionization chamber measurements in homogeneous phantom (Cheese phantom) for the different treatment plans.

	*Target (22 points)*	*OAR (27 points)*
Mediastinum	-0.5 ± 1.6%	0.5 ± 2.7%

Single metestasis	1.9 ± 1.5%	1.3 ± 3.1%

Multipla metastasis	0.8 ± 1.0%	1.1 ± 2.5%

Average	0.7 ± 1.2%	1.0 ± 0.4%

Percentage deviation between measured and calculated dose, for target and OAR point measurements.

Film data (EDR2 and EBT2) were analyzed in two ways: first, with a qualitative comparison of dose profiles; second by a quantitative gamma index analysis.

In Table 2 the percentage of points with gamma values ≤ 0.7, 1.0 and 1.5 were reported for different gamma index criteria, for both EDR2 and EBT2 films and for the three anatomical regions. Excellent agreement was also found for planar dose distributions: on average more than 97% of points passed the gamma test (γ ≤ 1) for EDR2 films with a 3%-3 mm criteria; a slightly worse, but acceptable agreement (94%) was found for EBT2 films; however, this value significantly increases using 4%-3 mm and 4%-4 mm criteria: 95.7% and 98% respectively.

**Table 2:** Gamma analysis distribution for the different treatment plans in homogeneous phantom (Cheese phantom) for different acceptance criteria, both for EDR2 and EBT2 film.

			*EDR2 film*							*EBT2 film*					
	**3%-3 mm**			**4%-3 mm**			**3%-3 mm**			**4%-3 mm**			**4%-4 mm**		

	***0.7***	***1***	***1.5***	***0.7***	***1***	***1.5***	***0.7***	***1***	***1.5***	***0.7***	***1***	***1.5***	***0.7***	***1***	***1.5***

Mediastinum	85	**96**	99	94	**98**	99	72	**85**	94	78	**90**	98	88	**95**	99

Single met.	96	**98**	100	97	**99**	100	95	**98**	100	97	**99**	100	98	**99**	100

Multipla met.	96	**99**	100	99	**100**	100	95	**99**	100	98	**99**	100	99	**100**	100

Percentage of points with *γ* value ≤ 0.7, 1 and 1.5 was reported.

### 3.2 Heterogeneous phantom

Table 3 shows the average percentage discrepancy between ion chamber measurements and TPS calculation for each simulated treatment plan and separately for PTV and OARs.

**Table 3:** Ionization chamber measurements in inhomogeneous thorax phantom for the different treatment plans.

	*Target*	*OAR*
Mediastinum 1	-1.7%	0.7%

Mediastinum 2	2.6%	-1.1%

Single metastasis	-2.1%	0.8%

Multipla metastasis	-2.9%	8.9%

**Average**	-1.0 ± 2.5%	2.3 ± 4.5%

Percentage deviation between measured and calculated dose, for target and OAR point measurements

An average discrepancy equal to -1% (1SD = 2.5%) and 2.3% (1SD = 4.5%) was found for target and OARs respectively. The worst agreement (-3% for PTV and around 9% for OARs) was found for multiple metastasis, probably due to the more stressed modulation applied in the irradiation.

**Figure 3:**
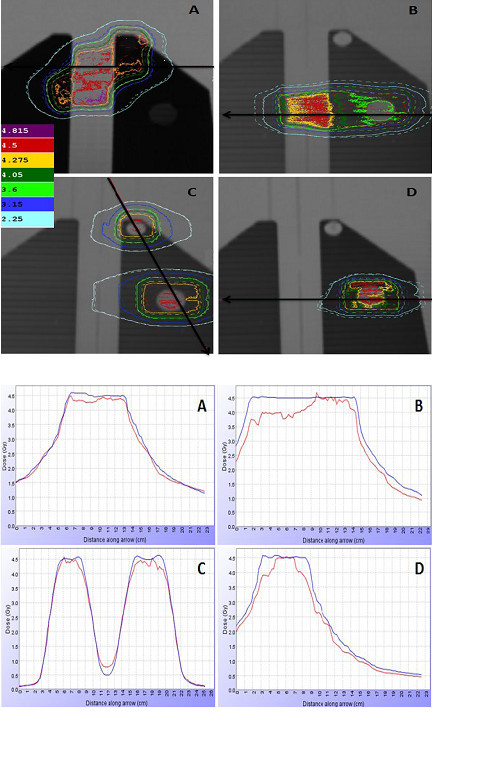
**Planar comparison between calculated and measured dose distributions with EDR2 films**. **3a) **Coronal isodoses comparison for mediastinum targets (A-B), multiple metastases (C) and single metastasis (D) plans in heterogeneous thorax phantom. The calculated distribution is identified by solid lines and the measured (EDR2 films) by dashed line. **3b) **Measured (red) and calculated (blu) dose profiles comparison for mediastinum targets (A-B), multiple metastases (C) and single metastasis (D).

**Figure 4:**
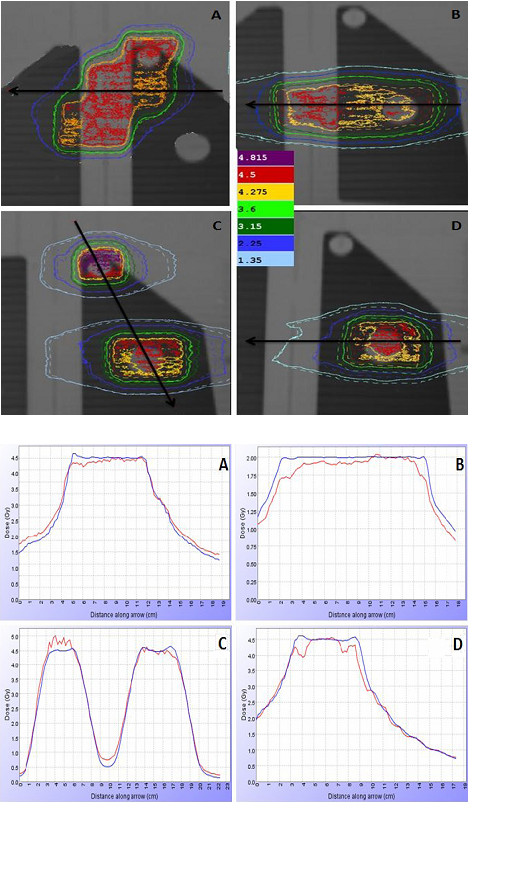
**Planar comparison between calculated and measured dose distributions with EBT2 films**. **4a) **Coronal isodoses comparison for mediastinum targets (A-B), multiple metastases (C) and single metastasis (D) plans in heterogeneous thorax phantom. The calculated distribution is identified by solid lines and the measured (EBT2 films) by dashed line. **4b) **Measured (red) and calculated (blu) dose profiles comparison for mediastinum targets (A-B), multiple metastases (C) and single metastasis (D).

Good agreement was qualitatively reported in Figure 3 and 4 where the comparison between measured and calculated isodoses (Figure 3a and 4a) and dose profiles (Figure 3b and 4b) was shown in a coronal plane for all four simulated treatment plans, both for EDR2 (Figure 3) and EBT2 (Figure 4)

In table 4 and 5 the percentage of points with gamma values ≤ 0.7, 1.0 and 1.5 were reported for several acceptable dose/distance criteria, respectively for EDR2 (Table 4) and EBT2 films (Table 5) and for the three anatomical regions, by separately considering the results for two different films, with film1 placed between the second (homogeneous) and the third slab (lung region) and film 2 placed between the third and the fourth slabs (lung/lung region).

**Table 4:** Gamma analysis distribution for the different treatment plans in inhomogeneous thorax phantom for different acceptance criteria, for EDR2 films.

	*EDR2 film*								
	**3%-3 mm**			**4%-4 mm**			**5%-4 mm**		

	***0.7***	***1***	***1.5***	***0.7***	***1***	***1.5***	***0.7***	***1***	***1.5***

***Med. 1(film 1)***	91	**96**	99	95	**98**	100	96	**98**	100
***Med. 1 (film2)***	92	**96**	98	94	**97**	100	95	**97**	100

***Med. 2(film 1)***	89	**91**	93	90	**93**	95	91	**93**	95
***Med. 2 (film2)***	81	**89**	94	84	**91**	94	87	**93**	94

***Single Met. (film 1)***	88	**96**	97	92	**96**	98	94	**97**	99
***Single Met. (film 2)***	88	**93**	97	91	**95**	99	93	**96**	99

***Multipla Met. (film 1)***	93	**95**	98	93	**96**	98	96	**97**	98
***Multipla Met. (film 2)***	85	**93**	99	89	**96**	100	92	**97**	100

Percentage of points with γ value ≤ 0.7, 1 and 1.5 was reported, separately for film 1 and film2.

**Table 5:** Gamma analysis distribution for the different treatment plans in inhomogeneous thorax phantom for different acceptance criteria, for EBT2 films.

	*EBT2 film*								
	**3%-3 mm**			**4%-4 mm**			**5%-4 mm**		

	***0.7***	***1***	***1.5***	***0.7***	***1***	***1.5***	***0.7***	***1***	***1.5***

***Med. 1(film 1)***	96	**98**	100	86	**99**	100	90	**100**	100
***Med. 1 (film2)***	70	**86**	92	83	**90**	95	87	**92**	96

***Med. 2(film 1)***	82	**93**	99	91	**98**	100	94	**99**	100
***Med. 2 (film2)***	85	**91**	94	90	**93**	97	91	**94**	99

***Single Met. (film 1)***	88	**95**	98	94	**97**	99	95	**98**	100
***Single Met. (film 2)***	80	**91**	98	89	**97**	100	92	**98**	100

***Multiple Met. (film 1)***	89	**95**	98	94	**98**	100	96	**98**	100
***Multiple Met. (film 2)***	66	**81**	92	78	**89**	96	82	**91**	97

Percentage of points with γ value ≤ 0.7, 1 and 1.5 was reported, separately for film 1 and film2.

For EDR2 films, 95% of points passed the gamma test (γ ≤ 1) with 4%-4 mm criteria, with slightly better results for film 1 (95.7% vs 94.7%). However, even with 3%-3 mm criteria the results were acceptable: 93.5% of points with γ ≤ 1 and only 3% of points with γ ≥ 1.5.

Comparable results were found for EBT2 films where on average 95% of points satisfy the 4%-4 mm criteria; the percentage of points with γ ≤ 1 was 98% and 92% for film 1 and film 2 respectively. Slightly worse results were found with 3%-3 mm criteria, where on average 91% of points have γ ≤ 1 have, with 95% of points for film 1 and around 87% of points for film 2.

## 4. Discussion and Conclusions

The Helical Tomotherapy treatment planning system uses a relatively accurate collapsed cone convolution/superposition algorithm for dose calculation and, as with other non -Monte Carlo algorithms, charged particle equilibrium is assumed in the dose calculation. For this reason we can expect inaccuracy in predicting dose distribution in the presence of significant inhomogeneities in patient geometry where this assumption is not satisfied. The dose distribution accuracy of the HT TPS was then tested in case of low density lung lesions.

Before the validation of the dose calculation algorithm in inhomogeneous media, the agreement between measured and calculated dose distributions for lung treatments was verified in a homogeneous phantom. Excellent agreement was found for point dose measurements with most of the data within ± 2%; an average percentage discrepancy equal to 0.85% (1SD = 0.5%) was estimated by considering all the points, both in PTV and in OAR regions. Good agreement (3%- 3 mm criteria) was also found for planar dose distributions, with 97% and 94% of points with γ ≤ 1, for EDR2 and EBT2 films respectively. The slightly worse results found with EBT2 could be probably correlated with the inaccuracy of the correction matrix applied to manage light scattering and non-uniform response of scanner lamp. The results found with EDR2 are in agreement with data published by Thomas et al [26], where the treatment plans of ten patients (head-neck, prostate, brain, bone metastasis) planned and treated with helical Tomotherapy were checked. An average point dose discrepancy of -1.3% was reported by con sidering high dose (-0.5 ± 1.1%), low dose (-2.4 ± 3.7%) and critical structure points (-1.1 ± 7.3%). By considering the 4 mm/3% criteria for EDR2 films, 92.6% and 99% of the measured points passed the test with γ ≤ 1 for the absolute and normalized planar dose distribution respectively; for these criteria our results were 99%.

The quality of the collapsed cone convolution algorithm implemented in the treatment planning of HT for homogeneous media was also confirmed in Zhao's paper [18], where a good agreement among MC simulations, TPS calculations, film and point dose measurements were reported and verified for a helical dose calculation performed on the cheese phantom. Point dose measurements in the PTV agree very well with TPS and MC calculations with deviations of 0.5% and 0.75%, respectively. TPS results agreed very well with MC simulation for 90%-10% Dmax dose levels; good agreement of 30%-90% isodose lines between calculation and film measurements were found for both TPS and MC results with acceptance criteria of 2%-2 mm, with a slightly larger discrepancy in regions with dose lower than 30% Dmax. Analysis of the gamma value distributions shows that for a 3%-3 mm criteria 100% of the points in the PTV pass the test both for MC and TPS calculations; for OARs around 90% and 93.5% of points agree with film measurements for MC and TPS calculations respectively. All the regions agree with film measurements, both for MC and TPS calculations, by considering a 5%-3 mm criteria.

In Zhao's paper [19] the accuracy of the CCS implemented in the HT treatment planning was evaluated against MC calculations and measurements in the CIRS anthropomorphic thorax phantom (lung density equal to 0.21 g/cm^3^), simulating a single helical treatment with a lung PTV containing water/tissue and part of the right lung. Considering points within 33% of the maximum dose, the average percentage discrepancy between ion chamber measurements and calculations was equal to - 1.4 ± 2.3% and 0.0 ± 0.81 for CCS HT and MC respectively. A wider difference was reported for planar dose distributions, where MC and TPS dose calculations were compared with relative dose distributions measured with EDR2 films. Using 3%-3 mm acceptance criteria, the MC agreed with measurements in around 90% of points, while the HT TPS is only 50%. With a clinically acceptable 5%-3 mm criterion, the MC agreed with film measurements in most of the phantom plane but the CCS HT failed in some of the high dose low density lung region, low dose boundary regions and high dose gradient regions, where TPS overestimates the PTV dose in the lung region and underestimates the dose in the lung-tissue interface.

Similar results were also reported in Sterpin's paper [20], where CCS HT dose distributions may result in an overestimation of the dose to PTVs encompassing lung tissues and/or air cavities. The reported results clearly show that the CCS algorithm predicts higher dose coverage of the target volume compared with MC calculations for small lung tumors; no significant differences were found for most of the other clinical cases.

In a recent paper of Chaudhari et al [17], HT calculated dose distributions were compared with the measurements in two treatment plans of oesophageal cancer; a cubic phantom with a mediastinum geometry was used and two different lung-equivalent materials (density equal to 0.28 and 0.16 g/cm^3^) considered. The agreement between point dose measured values and TPS was in most cases within 1% with an average discrepancy of -0.3 ± 0.8%. For tolerance criteria of 3%-3 mm, using gafchromic films, around 95% and 98% of points passed the test (γ ≤ 1), respectively for Balsa wood (0.16 g/cm^3 ^) and for the LN300 ((0.28 g/cm^3 ^), the two different media simulating the lung region. These both results were obtained by considering two film planes, both inserted between slabs of inhomogeneous low density media. No measurements were reported in the interface region between homogeneous and low density media. Our results for the inhomogeneous phantom (lung surrogate density equal to 0.04 g/cm^3^) and mediastinum clinical situations were worst: using the same criteria we found around 89% of points with γ ≤ 1, if we consider similarly to Chaudhari's paper only the film completely inserted in low density media (film2); better result were found (around 96% of pints) if we consider the film 1 inserted between homogeneous/inhomogeneous media.

In summary, based on the reported situations, the Tomotherapy TPS provides an accurate dose calculation with clinically acceptable results for the pre-treatment verification of all considered thoracic irradiations in (very) low density media. The results, both in terms of point measurements and in terms of profiles and planar dose distribution comparison, were in agreement with the acceptance criteria defined for IMRT verification. A direct comparison with Monte Carlo simulations should be investigated in the future

## Competing interests

The authors declare that they have no competing interests.

## Authors' contributions

GMC, PM, SB and RC carried out the study conception and design. VA and SB performed the measurements and data analysis. SB, VA and GMC drafted the manuscript. All authors read and approval the final manuscript.

## References

[B1] Papanikolaou N Battista JJ Boyer AL Kappas C Klein C Tissue inhomogeneity corrections for megavoltage photon beams Report of Task Group N°65 of the Radiation Therapy Committee of the American Association of Physicist in Medicine, AAPM Report N 2004 85

[B2] Sharpe MB Battista JJ Dose calculations using convolution and superposition principles:The orientation of dose spread kernels in divergent x-ray beams Med Phys 1993 20 1685 94 10.1118/1.596955 8309441

[B3] Vanderstraeten B Reynaert N Paelinck L Madani I Accuracy of patient dose calculation for lung IMRT: a comparison of Monte Carlo, convolution/superposition, and pencil beam computations Med Phys 2006 33 3149 58 10.1118/1.2241992 17022207

[B4] Francescon P Cora S Chiovati P Dose verification of an IMRT treatment planning system with the BEAM EGS4-based Monte Carlo code Med Phys 2003 30 244 57 10.1118/1.1538236 12607832

[B5] Jones AO Das IJ Comparison of inhomogeneity correction algorithms in small photon fields Med Phys 2005 33 766 76 10.1118/1.1861154 15839349

[B6] Seco J Evans PM Assessing the effect of electron density in photon dose calculations Med Phys 2006 33 540 52 10.1118/1.2161407 16532961

[B7] Mackie TR Scrimger JW Battista JJ A convolution method of calculating dose for 15 MV x-ray Med Phys 1985 12 169 77 10.1118/1.595774 4000075

[B8] Ahnesjo A Andreo P Brahme A Calculation and application of point spread functions for treatment planning with high energy photon beams Acta Oncol 1987 26 49 10.3109/02841868709092978 3109459

[B9] Ahnesjo A Collapsed cone convolution of radiant energy for photon dose calculation in heterogeneous media Med Phys 1989 16 577 92 10.1118/1.596360 2770632

[B10] Nisbet A Beange I Vollmar H Irvine C Morgan A Thwaites DI Dosimetric verification of a commercial collapsed cone algorithm in simulated clinical situations Radiother Oncol 2004 73 79 88 10.1016/j.radonc.2004.06.007 15465150

[B11] Chow JCL Leung MKK Van Dick J Variations of lung density and geometry on inhomogeneity correction algorithms: A Monte Carlo dosimetric evaluation Med Phys 2009 36 361 63 10.1118/1.3168966 19746796

[B12] Fogliata A Nicolini G Vanetti E Clivio A Winkler P Cozzi L The impact of photon dose calculation algorithms on expected dose distributions in lungs under different respiratory phases Phys Med Biol 2008 53 2375 90 10.1088/0031-9155/53/9/011 18421117

[B13] Mijnheer B Georg D Guidelines for the verification of IMRT Estro booklet No. 9 2008

[B14] Lu W Olivera GH Chen ML Reckwerdt PJ Mackie TR Accurate convolution/superposition for multi -resolution dose calculation using cumulative tabulated kernels Phys Med Biol 2005 50 655 80 10.1088/0031-9155/50/4/007 15773626

[B15] Mackie TR Balog J Ruchala K Shepard D Tomotherapy Semin Radiat Oncol 1999 9 108 17 10.1016/s1053-4296(99)80058-7 10196402

[B16] Liu HH Mackie TR McCullough EC Correcting kernel tilting and hardening in convolution/superposition dose calculations for clinical divergent and polychromatic photon beams Med Phys 1997 34 2070 6 10.1118/1.597960 9394280

[B17] Chaudhari SR Pechenaya OL Goddu SM Mutic S Rangaraj D The validation of tomotherapy dose calculation in low-density media Phy Med Biol 2009 54 2315 22 10.1088/0031-9155/54/8/004 19305040

[B18] Zhao Y Mackenzie M Kirby C Fallone BG Monte Carlo calculation of helical tomotherapy dose delivery Med Phys 2008 35 3491 3500 10.1118/1.2948409 18777909

[B19] Zhao Y Mackenzie M Kirby C Fallone BG Monte Carlo evaluation of treatment planning system for tomotherapy in an anthropomorphic heterogeneous phantom and for clinical treatment plans Med Phys 2008 35 5366 74 10.1118/1.3002316 19175096

[B20] Sterpin E Salvat F Olivera G Vynckier S Monte Carlo evaluation of the convolution/superposition algorithm of hi-art tomotherapy in heterogeneous phantoms and clinical cases Med Phys 2009 36 1566 75 10.1118/1.3112364 19544772

[B21] Cheung T Butson MJ Yu PK Post-irradiation colouration of Gafchromic EBT radiochromic film Phys Med Biol 2005 50 N281 N285 10.1088/0031-9155/50/20/N04 16204869

[B22] Menegotti L Delana A Martignano A Radiochromic film dosimetry with flatbed scanners: A fast and accurate method for dose calibration and uniformity correction with single film exposure Med Phys 2008 35 3078 85 10.1118/1.2936334 18697531

[B23] IAEA Absorbed dose determination in external beam radiotherapy. An International Code of Practice for Dosimetry Based on Standards of Absorbed Dose to Water Technical Reports Series 2000 398

[B24] Low D Harms W Mutic S Purdy H A technique for the quantitative evaluation of dose distributions Med Phys 1993 20 1709 19 10.1118/1.598248 9608475

[B25] Mijnheer B Quality assurance of treatment planning systems-practical examples for non-IMRT photon beams ESTRO Booklet No. 7 2004

[B26] Thomas SD Mackenzie M Field GC Syme AM Fallone BG Patient specific treatment verifications for helical tomotherapy treatment plans Med Phys 2005 32 3793 3800 10.1118/1.2134929 16475779

